# Clinical Profile of SARS-CoV-2-Infected Neonates

**DOI:** 10.7759/cureus.26298

**Published:** 2022-06-24

**Authors:** Rajesh K Kulkarni, Chhaya Valvi, Rahul Dawre, Uday Rajput, Rema Nagpal, Isha Deshmukh, Pragathi Kamath, Richa Harwani, Ramya Srinivasarangan, Somendra Sonteke, Apoorva R, Savita Kamble, Shilpa Naik, Ramesh Bhosale, Rakeesh Waghmare, Deepak Modi, Rahul Gajbhiye, Aarti A Kinikar

**Affiliations:** 1 Pediatrics, Postgraduate Institute, Yashwantrao Chavan Memorial Hospital, Pune, IND; 2 Pediatrics, Byramjee Jeejeebhoy (BJ) Government Medical College and Sassoon General Hospitals, Pune, IND; 3 Neonatology, Byramjee Jeejeebhoy (BJ) Government Medical College and Sassoon General Hospitals, Pune, IND; 4 Obstetrics and Gynaecology, Byramjee Jeejeebhoy (BJ) Government Medical College and Sassoon General Hospitals, Pune, IND; 5 Community Medicine, Grant Government Medical College, Mumbai, IND; 6 Reproductive Health, National Institute for Research in Reproductive Health, Mumbai, IND; 7 Reproductive Research, Indian Council of Medical Research (ICMR) - National Institute for Research in Reproductive Health, Mumbai, IND

**Keywords:** inflammation, sars-cov-2, newborn, clinical profile, mother to child transmission

## Abstract

Background

There are conflicting data on the mother-to-child transmission of severe acute respiratory syndrome coronavirus 2 (SARS-CoV-2) and few studies have described the clinical course of neonates infected with SARS-CoV-2.

Objectives

This study investigates the mother-to-child transmission rate and clinical profile of SARS-CoV-2-infected newborns.

Methods

Data on 304 newborns of 301 mothers with coronavirus disease 2019 (COVID-19) were prospectively collected and analyzed. Reverse transcription-polymerase chain reaction (RT-PCR) determined the presence of SARS-CoV-2 in the placenta, umbilical cord stump, and nasopharyngeal swabs collected within 24h of birth. Clinical and laboratory data of SARS-CoV-2-infected newborns was entered in a structured proforma.

Results

A total of 20 neonates (6.5%) were positive for SARS-CoV-2, of which 12 were positive only in the nasopharyngeal swab, four cases had the umbilical stump positive, three were positive in the placenta, and one case was positive in all the three specimens collected. Six of the 20 SARS-CoV-2-positive neonates developed severe symptoms. The SARS-CoV-2-positive symptomatic neonates required a more extended stay in hospital compared to their non-symptomatic infected counterparts.

Conclusions

A proportion of the babies born to SARS-CoV2-infected mothers tested positive and some of these newborns had severe symptoms.

## Introduction

The coronavirus disease 2019 (COVID-19) pandemic caused due to severe acute respiratory syndrome coronavirus 2 (SARS-CoV-2) continues to spread across the world. The primary route of transmission of the virus is via respiratory droplets and/or close contact between people with family clustering [1].

A major concern of SARS-CoV-2 infection in pregnancy is its possible effects on newborns. There is now growing evidence demonstrating that a proportion of infants infected with SARS-CoV-2 have serious presentations resembling multisystem inflammatory syndrome [2-3]. In the context of mother-to-child transmission, some studies have reported that there were no SARS-CoV-2-positive infants born to a cohort of mothers with COVID-19 while others have reported an incidence of as high as 10% [4-8]. A metanalysis revealed that of 330 infants born to COVID-19 mothers, nine (2.7%) tested positive for SARS-CoV-2 [8]. In another metanalysis, 27 out of 936 neonates (3.2%) born to COVID-19 mothers were found to be positive for SARS-COV-2 in the nasopharyngeal swab [9]. Prior studies have suggested that neonatal exposure to COVID-19 may include adverse outcomes, such as preterm birth, and respiratory distress, among others [10].

Herein, we report the mother-to-child transmission rate and clinical features of 20 SARS-CoV-2-positive neonates.

## Materials and methods

The study was approved by the Institutional Ethics Committee of Byramjee Jeejeebhoy (BJ) Government Medical College and Sassoon General Hospitals, Pune (BJGMC/IEC/Pharmac/ND/-Dept 0420080-080), and informed consent was obtained from the participants. The PregCovid registry (https://pregcovid.com/) prospectively collects data on mothers with COVID-19 and their neonates.

Study site

The study was conducted at BJ Government Medical College and Sassoon Hospital, Pune, India, which is one of the participating centers of the PregCovid registry network. The study period was from July 1, 2020, to December 31, 2021.

Inclusion criteria

All pregnant women admitted to the hospital presenting in labor or likely to deliver in five days were tested for COVID-19 and those who tested positive and delivered were included in the study.

Study procedures

Maternal nasopharyngeal swabs were collected either on an outpatient basis or after admission. Upon delivery, pieces of the placenta from the site of cord insertion and umbilical cord stump were collected. The neonatal nasopharyngeal swab was collected within 24h of birth and 24h after the first swab to confirm the result. The tissues and swabs collected in a viral transport medium were sent to the microbiology lab of the hospital where reverse transcription-polymerase chain reaction (RT-PCR) for SARS-CoV-2 was done using primers specific for the E and ORF gene (Applied Biosystems AB 7500 fast Dx; Waltham, Massachusetts). A sample was deemed positive if both primers yielded a positive report as per the kit cutoffs.

Data collection and statistical analysis

**Figure 1:**
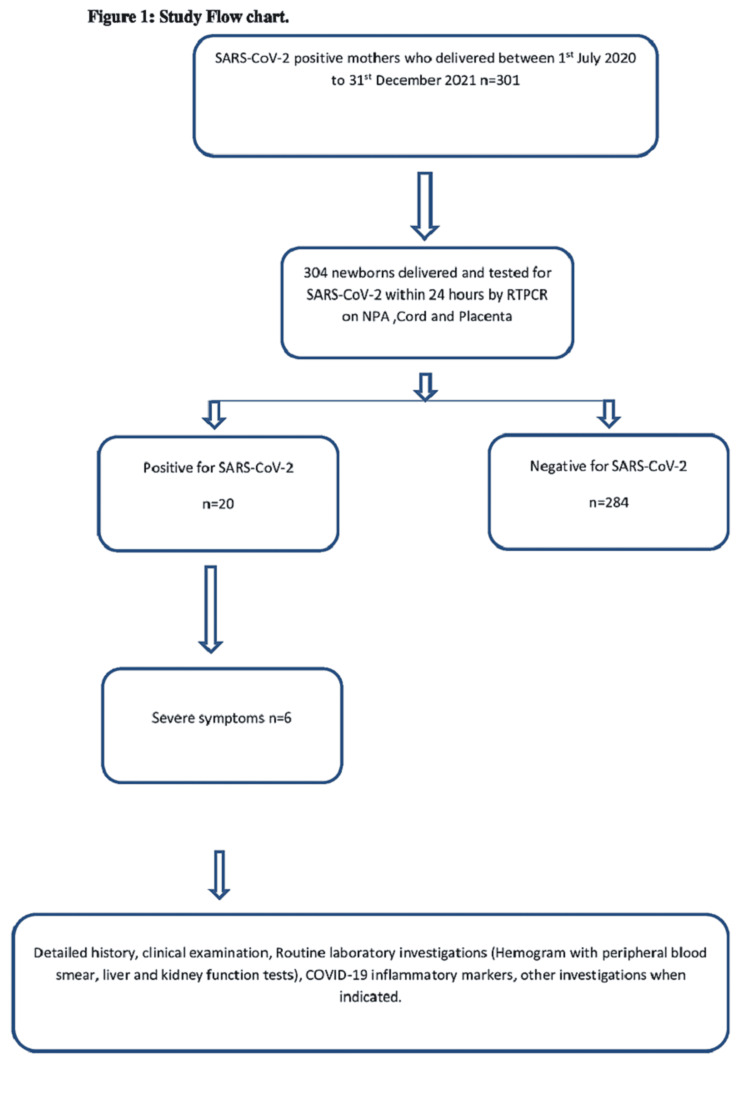
Study flow chart

Irrespective of the symptomatic status, COVID-19-positive pregnant women were delivered in a separate, designated labor room. Data of maternal age, COVID vaccination status, period of gestation, history of contact, symptoms, associated comorbidities, mode of delivery, and details of the newborn were prospectively recorded in the PregCovid registry and extracted thereafter. Figure 1 shows the study flow chart.

## Results

During the study period, 301 women with a laboratory-confirmed diagnosis of SARS-CoV-2 via a nasopharyngeal swab were enrolled. The median age of the mothers was 24 years (IQR 21-26). Twelve mothers had preeclampsia, and 11 had moderate anemia (Hb 7-10 gm/dl). Sixty point five percent (60.5 %, n=182) of the pregnant women delivered vaginally while 34.8% (n=105) delivered via cesarean section and 4.8% (n=14) required an instrumental vaginal delivery. Fetal distress was found in eight cases and there were six cases of prolonged rupture of the membrane (greater than 18 hours). Forty-two mothers (13.9%) were symptomatic while 259 mothers (86%) were asymptomatic. Among the symptomatic mothers, 71% had mild disease, 16% had moderate while 12% had severe disease requiring ICU admission. There were three maternal deaths (1%). There were 298 singleton and three twin deliveries resulting in 304 neonates. Except for one, all the newborns had normal Apgar scores and were isolated in a separate neonatal intensive care unit (NICU)/neonatal isolation ward. There were 166 male and 138 female neonates. The median gestational age of the neonates was 37.2 weeks (IQR 2.1), and the median birth weight was 2412.5 g (IQR 787.5). Of the 304 neonates, 7.4% were born preterm (<37 weeks of gestation) and 13.8% had low birth weight (<2500g). Acute respiratory distress was observed in 5.2% of neonates while 4.2% had oxygen saturation (SpO2) below 90%. Eighteen neonates (5.9%) required NICU admission for various reasons. There were no neonatal deaths. The symptomatic SARS-CoV-2 positive neonates had a longer stay in NICU compared to asymptomatic SARS-CoV-2-infected neonates (Table 1).

**Table 1:** Presentation of mothers with COVID-19 and their neonates The 95% confidence interval for a proportion was calculated using the UCSF sample size calculator: https://sample-size.net/confidence-interval-proportion/

	Numbers	Percentage	95% confidence interval
Maternal Presentation			
SARS-CoV-2 positive	301		
Symptomatic	42	13.9	0.10-0.18
Asymptomatic	259	86.0	0.81-0.89
Mild	30	10.0	0.55-0.84
Moderate	7	2.3	0.06-0.31
Severe	5	1.6	0.03-0.25
Deaths	3	1.0	0.002-0.028
Singletons	298	99.0	0.97-0.99
Twins	3	0.9	0.002-0.02
Vaginal delivery	182	60.4	0.54-0.66
Instrumented aided vaginal delivery	14	4.6	0.02-0.07
Caesarean Section	105	34.8	0.29-0.40
Neonatal presentations			
Total neonates	304	-	-
SARS-CoV-2 positive	20	6.5	0.04-0.09
Premature baby(<3 7 weeks)	23	7.5	0.04-0.11
Required resuscitation	8	2.6	0.01-0.05
Low birth weight (<2500 gm)	42	13.8	0.10-0.18
Spo2 below 90%	13	4.2	0.02-0.07
Acute respiratory distress	16	5.2	0.03-0.08
NICU admission	18	5.9	0.03-0.09
Deaths	0	0	-
Days of NICU stay (mean)			
SARS-CoV-2 positive symptomatic newborn	17	-	-
SARS-CoV-2 positive asymptomatic newborn	5	-	-

Twenty neonates (6.5%) born to COVID-19 mothers (of which two mothers had a severe illness and eight were symptomatic) were positive for SARS-CoV-2. Among the 20 SARS-CoV-2-positive neonates, 12 were positive only in the nasopharyngeal swabs (60%), four cases had only the umbilical stump positive (20%), three were positive in the placental tissue (15%) while one case had all the three specimens positive (5%).

Six of the 20 SARS-CoV-2-positive newborns developed signs and symptoms like poor feeding and icterus. All six babies had evidence of inflammation in the form of raised ferritin, interleukin-6 (IL-6), and positive C-reactive protein (Table 2). The blood cultures of all six babies were negative and chest radiographs were normal except in Case 2 where the chest radiograph showed diffuse bilateral para hilar infiltrates. All six received intravenous antibiotics for at least seven days and immunoglobulins along with supportive care (Table 2).

**Table 2:** Individual data of SARS-CoV-2-positive and symptomatic neonates ND: not done, IVIG: intravenous immunoglobulin, HIE: hypoxic-ischemic encephalopathy

Case Number	1	2	3	4	5	6
Maternal age (years)	25	26	24	28	20	30
Gestational age when Covid-19 positive (weeks)	39	30.4	38.2	37.2	37	39
Maternal presentation	Mild symptomatic	Asymptomatic	Mild symptomatic	Asymptomatic	Asymptomatic	Asymptomatic
Maternal Co-morbidities	None	None	None	None	None	None
Mode of Delivery	Cesarean	Cesarean	Assisted vaginal	Cesarean	Vaginal	Cesarean
Samples positive for SARS-CoV-2	Nasopharyngeal swab	Nasopharyngeal swab	Nasopharyngeal swab, Cord stump, Placenta	Nasopharyngeal swab	Cord stump	Placenta
Birth weight in grams	3250	1400	3200	3100	2200	2400
Clinical course	Poor feeding, lethargy, mild tachypnea	Tachypnea requiring oxygen, lethargy	Fever, Poor feeding, Hyperbilirubinemia	HIE stage 2, Bilateral Pneumothorax	temperature, instability, poor feeding, tachypnea	Poor feeding, lethargy, tachypnea
NICU stay (days)	8	24	21	31	10	10
Serum IL-6 (Normal range 0-20.9 pg/ml)	ND	ND	43	54	51	65
Serum Ferritin (Normal range 36-391 ng/ml)	463	431	774	763	470	694
C-reactive protein (Normal range 0-10 mg/L)	18	16	22	24	18	21
Treatment given	Oxygen, Antibiotics, IVIG	Oxygen, Antibiotics, IVIG	Oxygen, Antibiotics, IVIG, Lopinavir Ritonavir	Mechanical ventilation, Antibiotics, IVIG	Oxygen, IV Antibiotics, IVIG	Oxygen, IV Antibiotics, IVIG

## Discussion

SARS-CoV-2 infection during pregnancy is a matter of great concern, as the mothers are in a physiologically and immunologically compromised state, and there is a risk of mother-to-child transmission. Extending the previous results (Waghmare et al.), herein we observed that less than 15% of SARS-CoV-2-infected mothers are symptomatic, and most of them had a mild presentation. However, it is important to note that in our study, one in twenty-five women developed severe disease with one in sixty women requiring ICU care and one in hundred pregnant women with COVID-19 ultimately died. This number of pregnant women with COVID-19 requiring ICU admission or death is higher than those without COVID-19 or those in the pre-pandemic period (not shown). A similar increased rate of severe disease and maternal mortality has been recently reported in the American population, suggesting that pregnant women should be considered a high-risk population for COVID-19 until proven otherwise [11]. It has been previously reported that infected women with twin gestations did not present with severe disease as compared to their singleton counterparts [12]. Our study had three twins and their mothers did not experience severe disease.

Maternal infections can have an adverse impact on newborn health and many viral infections increase the risk of preterm births [13]. In the case of SARS-CoV-2, some studies have shown an increased risk of preterm births [13-14]. In our study, the mean gestational age was 37+3 weeks and only 6.7% of babies were born preterm, which is lower than the observed numbers of preterm births in the non-pandemic time [15-16]. A lower than usual incidence of preterm birth is a consistent observation made in many Low and Low Middle-Income Countries (LMICs) during the COVID-19 pandemic [17]. Improved air quality during the lockdown, better hygienic practices, and social distancing reduced anxiety due to family attention and homestay, and reduced work pressures in working women are the possible explanations for reduced rates of preterm births [18]. However, the possibility of increased home deliveries during the lockdown period could lead to the under-reporting of preterm births in a tertiary referral center. More studies will be required to address this finding.

Newborns acquiring SARS-CoV-2 infection are thought to be relatively uncommon [19-21]. However, in many instances, whether the infection is acquired in utero or postnatally from the mother is unclear. Consistent with our findings, a recent meta-analysis of 936 neonates born to mothers with COVID-19 revealed a pooled proportion of 3.2% for SARS-CoV-2 positivity in the nasopharyngeal swabs [9]. We could not do testing as per the criteria laid down for the diagnosis of transplacental or intrapartum transmission due to a lack of resources [22-23].

Although it is presumed that COVID-19 may not be very severe in the neonatal population, it is important to note that six out of 20 SARS-CoV-2 infected neonates developed symptoms and required long NICU admission as compared to their uninfected counterparts born to infected mothers. Multisystem inflammatory syndrome (MIS) is reported to be a complication of SARS-CoV-2 infections in neonates [24]. In our study, all the six neonates who developed symptoms had conditions of inflammation. Although we have not investigated other systemic markers of PMIS, our data suggest that a subset of neonates born to COVID-19 mothers will require medical attention although there were no deaths.

The determinants of symptomatic SARS-CoV-2 in newborns are hard to predict. Of the six symptomatic neonates, two were born to symptomatic mothers while four mothers were asymptomatic. Also, the mode of delivery does not seem to show any relationship as four newborns were delivered by cesarean section, and two were delivered vaginally (one requiring assistance).

Although previous studies have shown symptomatic presentations in newborns with nasopharyngeal swabs, in our study, we found that one full-term neonate who was severely symptomatic was positive only in the placenta. Furthermore, there was no relationship between the site of infection and length of stay in NICU or clinical presentations. Thus, our study suggests that continued vigilance is needed as maternally acquired SARS-COV-2 can potentially cause serious complications in the newborn and the babies must be clinically monitored for adverse outcomes. Nevertheless, this study provides clear evidence that pregnant patients with COVID-19 are likely to transmit the virus to the newborn, and these infants are at an elevated risk of poor outcomes.

Our study had a few limitations. Being a single-centered study, the numbers were small. Also, we could not do testing to establish vertical transmission per the guidelines due to the unavailability of some tests and the pandemic situation putting a huge workload on treating staff. Also, some of the symptomatic neonates had other conditions that could have resulted in severe symptoms (like perinatal asphyxia and pneumothorax), and it was not possible to say how much the SARS-CoV-2 infection contributed to the severity of symptoms in these neonates.

## Conclusions

This single-centered study estimates that the SARS-COV-2 positivity rate in neonates born to mothers with COVID-19 is 6.5% (one in every 15 newborns). Further, a proportion of these babies would have a severe symptomatic presentation and may require prolonged hospitalization and continuous clinical monitoring. Few babies may need intensive care admission. Most of the babies born to SARS-COV-2-positive mothers in our study were term babies.

Studies in diverse populations directed toward early-onset COVID-19 disease in neonates are required to guide patients and help physicians and policymakers develop disease management strategies.
